# A Metabolomic Analysis to Assess the Responses of the Male Gonads of *Mytilus galloprovincialis* after Heavy Metal Exposure

**DOI:** 10.3390/metabo13121168

**Published:** 2023-11-22

**Authors:** Gennaro Lettieri, Carmela Marinaro, Carlo Brogna, Luigi Montano, Martina Lombardi, Alessio Trotta, Jacopo Troisi, Marina Piscopo

**Affiliations:** 1 Department of Biology, University of Naples Federico II, Via Cinthia, 21, 80126 Naples, Italy; 2 Department of Research, Craniomed Group Facility S.r.l., 20091 Bresso, Italy; 3 Andrology Unit and Service of LifeStyle Medicine in Uro-Andrology, Local Health Authority (ASL) Salerno, 84084 Salerno, Italy; 4 Theoreo S.r.l.—Spin-off Company, University of Salerno, 84084 Salerno, Italy

**Keywords:** gonads, metabolomics, heavy metals, *Mytilus galloprovincialis*

## Abstract

In recent years, metabolomics has become a valuable new resource in environmental monitoring programs based on the use of bio-indicators such as *Mytilus galloprovincialis*. The reproductive system is extremely susceptible to the effects of environmental pollutants, and in a previous paper, we showed metabolomic alterations in mussel spermatozoa exposed to metal chlorides of copper, nickel, and cadmium, and the mixture with these metals. In order to obtain a better overview, in the present work, we evaluated the metabolic changes in the male gonad under the same experimental conditions used in the previous work, using a metabolomic approach based on GC-MS analysis. A total of 248 endogenous metabolites were identified in the male gonads of mussels. Statistical analyses of the data, including partial least squares discriminant analysis, enabled the identification of key metabolites through the use of variable importance in projection scores. Furthermore, a metabolite enrichment analysis revealed complex and significant interactions within different metabolic pathways and between different metabolites. Particularly significant were the results on pyruvate metabolism, glycolysis, and gluconeogenesis, and glyoxylate and dicarboxylate metabolism, which highlighted the complex and interconnected nature of these biochemical processes in mussel gonads. Overall, these results add new information to the understanding of how certain pollutants may affect specific physiological functions of mussel gonads.

## 1. Introduction

Metabolites are the substrates and products of metabolism that drive essential cellular functions and are produced directly by the host organism, as well as by xenobiotic, dietary, and other exogenous sources [1]. Metabolomics consists of the comprehensive study of metabolites, and since the metabolic profile is directly or indirectly involved in every aspect of cellular function, metabolomics is considered a mirror of the phenotype of each cell [2]. All changes in the proteome and transcriptome are in fact reflected in the final metabolic profile. Over the past decades, many metabolomic studies have been conducted to evaluate and explore potential mechanisms of toxicity and to identify toxicological biomarkers [3]. Heavy metals are among the toxic elements that contribute to the pollution of the marine environment [4]; in fact, their ability to bioaccumulate in different environmental compartments, in animals, and in plants, and to pass through the food chain and reach people, is a relevant problem [5]. That is because heavy metals can bind to crucial biomolecules like proteins and enzymes, altering enzyme activity and ultimately leading to cell dysfunction. In addition, heavy metals have the ability to produce reactive oxygen species (ROS), resulting in the oxidative damage of cellular components including proteins, lipids, and DNA. The reproductive toxicity of heavy metals in marine invertebrates is an important issue to consider. This aspect has been the subject of some research in the literature, but the majority of studies have generally focused on the effects of single metals, e.g., in the case of *Mytilus galloprovincialis* (*M. galloprovincialis*) [6,7]. Because the various heavy metals may act in a synergetic or antagonistic manner, enhancing or reducing individual effects, it is particularly interesting to assess their effects on the sentinel organisms employed in bio-monitoring programs. Benthonic organisms, in particular bivalve mollusks, are widely used, especially for the monitoring of the coastal environment [8,9,10]. This is because mussels are sedentary organisms that feed on filter-feeding substances, tending to bioaccumulate and magnify pollutants. Therefore, these organisms are particularly useful to determine the long-term accumulation of pollutants in the environment. However, environmental contamination is a persistent problem of particular relevance to humans [11]. Although many methods have been used to better understand and minimize this problem, it remains elusive. The consequences of these environmental risks are global, so new methods must be developed to protect human health and the environment [12]. New approaches and opportunities for environmental management have emerged with the development of molecular techniques such as transcriptomics, proteomics, and related omics approaches. Such techniques provide new and deeper insights into the functioning of pollutants, which can help to better understand their environmental risks [13]. Among these techniques, metabolomics is proving to be the most promising method for assessing the response of different organisms to environmental pressures [14]. This approach can identify metabolites implicated in biochemical processes and provide insight into pathways perturbed by environmental stressors such as pollutants, starvation, and pathogens [15,16]. This is achieved by assessing changes in the levels of metabolites, precursors, and products of enzyme activity and attempting to relate these changes to biological function and/or regulation. Research studies in marine bivalve mollusks have reported the use of metabolomics for the characterization of metabolic responses and toxicity of specific contaminants [17,18,19]. In our previous work, we evaluated the metabolomic pattern of *M. galloprovincialis* spermatozoa after exposure to three different heavy metals and their mixture, and we found several altered metabolites implicated in important sperm functions [20]. These results support the idea that omics approaches can be used as methods to search for new biological markers. For this reason, in the present work, we continue to explore the metabolomic pattern in *M. galloprovincialis* in a non-filtering tissue, in particular, the gonadal tissue after exposure to the same heavy metals and their mixture tested in the previous work [20]. The concentrations used for the study were cadmium 1.5 µM, copper 15 µM, and nickel 15 µM, because they were tested in our previous investigations and showed the most noticeable effects on the *M. galloprovincialis* reproductive system by other experimental approaches [21,22]. All doses are subtoxic. Therefore, the aim of this work is to evaluate the possible alterations in the level of metabolites as promising indicators of the response of gonadal tissue, crucial for reproduction, to external stresses such as the presence of heavy metals.

## 2. Materials and Methods

### 2.1. Ethical Statement

This study has been carried out on the marine invertebrate *M. galloprovincialis* (Lamarck, 1819). This species is not under the protection of any environmental authority in Italy. This study was carried out in strict compliance with European legislation (Directive 2010/63) and with Italian legislation (Legislative Decree 116/1992) on the care and use of animals used for scientific purposes.

### 2.2. Exposure of Mussels

*M. galloprovincialis* specimens provided by Eurofish Napoli S.R.L. Bacoli (Campania region) were selected to study the effects of heavy metal exposure on male gonads. The specimens had an average shell size of 4.95 ± 0.17 cm and were of mixed sex. The mussels were acclimated at 18 °C ± 1 °C in plastic laboratory tanks before exposure to heavy metals. The mussels were exposed to different doses of metals, in accordance with the report by Lettieri et al. (2022) [22]: 15 µM, 15 µM, and 1.5 µM of chlorides of nickel, copper, and cadmium, respectively, and the mixture of these metals in these doses. Briefly, plastic tanks measuring 36 × 22 × 22 cm were used to expose *M. galloprovincialis* specimens. The tanks contained 6 L of artificial sea water (ASW) at 33‰. Each liter of ASW contained 29.2 g of NaCl, 0.60 g of KCl, 1.2 g of MgCl_2_, 0.20 g of NaHCO_3_, and 1.08 g of CaCl_2_. A total of 15 mussels were placed in each tank for 24 h at a temperature of 18 ± 1 °C. The oxygen level and temperature of the tanks were checked periodically, and the water and metal salts were changed after 12 h. The experiments were carried out in February and March 2023. A tank containing only ASW, as described by Lettieri et al., 2019 [23], was used as a control (unexposed mussels).

### 2.3. Processing and Sampling of Spermatozoa

After exposure to the various heavy metals tested, the mussels were opened with a knife. This operation left the soft tissues intact. The gonads were left in a tube containing 500 µL of ASW for 5 min at 16 °C. This facilitated the release of gametes. A 40× light microscope was used to determine the sex of the mussels. Male gonads were then left in the same tube for 1 h, after adding another 500 µL of AWS to allow the release of all spermatozoa, so that the analysis could then be performed on empty gonads.

#### 2.3.1. Metabolite Sample Processing: Extraction, Purification, and Derivatization

The process of extracting, purifying, and derivatizing the metabolome was conducted with the employment of the MetaboPrep GC kit, which is produced by Theoreo S.r.l. in Montecorvino Pugliano, Italy, with strict adherence to the manufacturer’s furnished guidelines. In summary, a homogenized portion of the 25 mg sample was scrupulously transferred into a microcentrifuge tube, accompanied by the suitable extraction solution and an internal standard. Subsequently, the tubes underwent robust agitation at a speed of 1250 rotations per minute (rpm) for a duration of thirty minutes. Following this, the resultant solution was subjected to centrifugation at a temperature of 4 °C and a high speed of 16,000 rpm for five minutes. From the resulting mixture, 200 µL of the unclouded supernatant was conveyed to a distinct microcentrifuge tube containing a purification solution. This new tube was briefly agitated at 1250 rpm for thirty seconds and, once more, subjected to centrifugation at 16,000 rpm and 4 °C. Following this step, 175 µL of the transparent supernatant was transferred to a glass vial and promptly frozen at −80 °C, where it was subsequently subjected to an overnight freeze-drying process. To prepare the freeze-dried samples for further analysis, the metabolites enclosed within underwent a two-phase derivatization procedure. Initially, 50 μL of methoxylamine hydro-chloride in pyridine was incorporated into the freeze-dried samples and agitated at 1200 rpm for a duration of ninety minutes. Subsequently, 25 µL of a derivatization solution, comprising N,O-Bis(trimethylsilyl)trifluoroacetamide (BSTFA) and trime-thylchlorosilane (TMCS), was introduced to the mixture. The vials were once again agitated at 1200 rpm for an additional ninety minutes. The resulting derivatized metabolites (75 µL) were then transferred to a vial insert with a capacity of 100 µL, facilitating their subsequent injection into an autosampler. Before injection into the GC-MS instrument, the vials were subjected to centrifugation for five minutes at 16,000 rpm at 4 °C.

#### 2.3.2. Gas Chromatography–Mass Spectrometry (GC-MS) Metabolomic Profiling

Subsequent to the preparation process, subsamples of 2 µL each were introduced into the GC-MS system, an instrument configuration consisting of a GC-2010 Plus gas chromatograph seamlessly coupled to a 2010 Plus single quadrupole mass spectrometer, crafted by Shimazu Corp (Kyoto, Japan). Chromatographic separation was achieved by employing a CP-Sil 8 CB fused silica capillary GC column, featuring dimensions measuring 30 m in length by 0.25 mm in diameter and a film thickness of 1.00 µm, sourced from Agilent (Agilent, J&W Scientific, Santa Clara, CA, USA), headquartered in Folsom, CA, USA. Helium was employed as the carrier gas, offering optimal conditions for this analytical endeavor.

The initial setting for the oven temperature was artfully established at 100 °C, a temperature maintained for a meticulous duration of 1 min. Following this initial phase, a gradual increase was implemented, raising the temperature at an exacting rate of 6 °C per minute until it reached its final temperature of 320 °C, a temperature precisely held for an additional 2.33 min. To ensure a consistent linear velocity of 39 cm/s, the gas flow rate was scrupulously adjusted, with the split flow adhering to a specific ratio of 5:1.

In the operation of the mass spectrometer, an electron impact mode was engaged, operating at an energy level of 70 electronvolts (eV). The mass spectrometer then executed an exhaustive scan analysis spanning the *m*/*z* range of 35–600, characterizing this scan with a scan velocity of 3333 atomic mass units per second (amu/s) and a solvent delay of 5 min, thus adhering to the highest standards of precision. The comprehensive GC program was characterized by a total duration of 40 min, a testament to the thoroughness and care taken in the analysis.

#### 2.3.3. Metabolite Identification

Metabolite identification procedures were executed, following the guidelines delineated by Troisi et al. [24,25,26]. To elucidate untargeted metabolites, an examination of each peak’s associated mass spectrum was conducted, cross-referencing it against the extensive NIST-2014 library compilation, conveniently hosted at the NIST facility in Gaithersburg, MD, USA. This comparative analysis was conducted with a maximum tolerance limit of 10 for the differential linear retention index. Furthermore, the search within the matching spectra library was held to a threshold of 85%, aligning with the exacting Level 2 identification standards defined by the Metabolomics Standards Initiative (MSI) [27], wherever feasible. Metabolites that failed to meet these stringent criteria were appropriately classified as “unknown,” in full accordance with MSI Level 4.

Signals per sample, which emerged through the fusion of gas chromatography and mass spectrometry, were judiciously excluded from further analysis if they met one of the following criteria: either they were conspicuously absent in less than 80% of the samples, or they were discerned in concentrations considered inadequate, or they exhibited less than optimal spectral quality, thereby rendering their metabolite identification a challenging endeavor.

Following this comprehensive analysis, a robust and consistent identification process successfully unveiled a grand total of 248 endogenous metabolites. To affirm the statistical significance of these identified metabolites in effectively distinguishing between different categories or classes, only those exhibiting a VIP score that surpassed the stringent threshold of 1.5 were chosen for confirmation through the utilization of independent analytical standards, aligning this verification with the rigorous MSI Level 1 criteria.

### 2.4. Statistical Analysis

#### 2.4.1. Profiling Animal Characteristics

The assessment of the animals’ characteristics involved a thorough statistical analysis conducted utilizing R, a dedicated programming language and environment tailored for the purpose of statistical computing and data visualization [28]. To assess the normality of the data distribution, the Shapiro–Wilk test was employed. In light of the data exhibiting a normal distribution, we proceeded to employ the one-way analysis of variance (ANOVA), complemented by Tukey’s post hoc test, for the purpose of conducting inter-group comparisons. The predetermined threshold for statistical significance, denoted as “alpha,” was firmly set at 0.05 in order to establish the statistical significance of the observed results.

#### 2.4.2. In-Depth Metabolomic Data Examination

The GC-MS metabolomic results were amalgamated into a matrix file, where values were delimited by commas. Subsequently, this file was imported into a specialized software solution, specifically, MetaboPredict^®^ from Theoreo S.r.l., Montecorvino Pugliano, Italy, for the purpose of conducting the ensuing statistical analysis.

Prior to the commencement of the analysis, the chromatographic data underwent a sequence of preprocessing steps. Data alignment was accomplished through the utilization of the parametric time warping algorithm [29]. Following this, essential processes such as peak detection, integration, and deconvolution were executed. The resulting chromatographic data were systematically structured in a tabular format, where each individual sample was thoughtfully represented as a row, while each metabolite was distinctly indicated as a column.

In the quest for data accuracy and consistency, a series of normalization procedures were also applied. These procedures encompassed both data transformation and scaling. Data transformation was facilitated through the employment of a generalized logarithmic transformation. In parallel, scaling was judiciously implemented using an auto-scaling approach, which encompassed mean-centering and standard deviation-based scaling for each variable (metabolite). Furthermore, an additional level of normalization was performed, considering the chromatographic peak area of the internal standard and the precise sample weight [30].

The analysis entailed the classification of the samples based on their respective treatments, resulting in the formation of five distinct classes: cadmium, copper, nickel, mix, and CTRL. To bolster the reliability of data analysis, the Synthetic Minority Over-sampling Technique (SMOTE) [31] algorithm was harnessed to generate five synthetic samples, one corresponding to each class.

The class separations underwent further exploration through the application of partial least squares discriminant analysis (PLS-DA), a supervised method that leverages multivariate regression techniques to uncover linear combinations of the original variables (X) capable of predicting class membership (Y). To assess the significance of class discrimination, a permutation test was also executed. During each permutation, a PLS-DA model was methodically constructed, involving the data (X) and permuted class labels (Y), while taking into consideration the optimal number of components, a determination made via cross-validation based on the original class assignments. Two distinct test statistics were employed to evaluate class discrimination. The first was grounded in prediction accuracy during training, while the second was based on the separation distance, calculated as the ratio between the sum of squares “Between” groups and the sum of squares “Within” groups (B/W-ratio). If the observed test statistic happened to fall within the distribution derived from permuted class assignments, the class discrimination was deemed statistically non-significant [32]. Variable importance in projection (VIP) scores were computed for each component within the PLS-DA analysis. VIP represents a weighted sum of squares of the PLS loadings, taking into account the extent of Y-variation explained in each dimension.

## 3. Results

**Figure 1:**
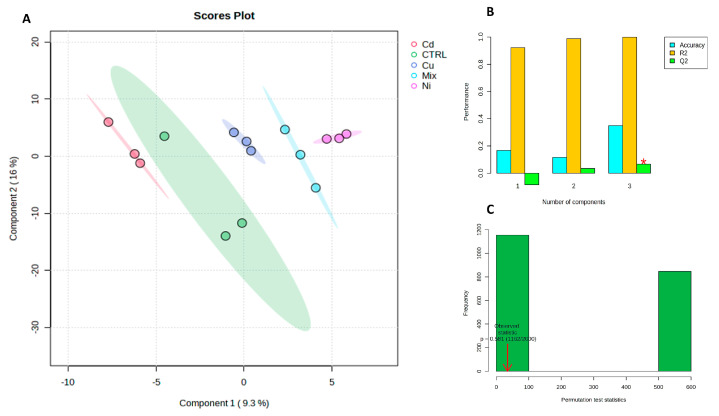
(**A**) Discriminatory analysis using partial least squares discriminant analysis (PLS-DA) to differentiate gamete samples of mussels subjected to various treatments: cadmium (red), copper (blue), mixed (cyan), nickel (violet), and control (green). Each axis is accompanied by the percentage of variance explained, indicated in parentheses. (**B**) illustrates the performance of the PLS-DA model for classification as the number of latent variables increases, with the optimal classifier marked by a red star. In (**C**), the results of the permutation test are presented, wherein models were constructed by randomly assigning class labels, and their performance was compared to the original model constructed with correct class assignments.

**Figure 2:**
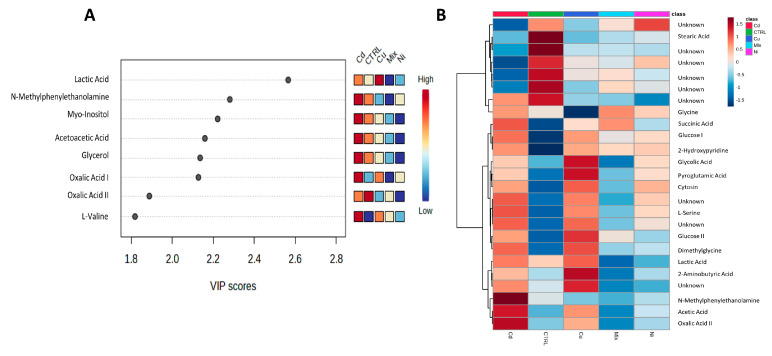
(**A**) Metabolites of significance in separating classes (VIP-score > 1.5). (**B**) Heatmap illustrating the concentrations of metabolites selected through ANOVA. The application of cluster analysis aided in identifying four clearly defined clusters of metabolites, characterized by their average concentration levels across the five classes.

The results were acquired through the examination of gonad tissue extracted from a set of ten mussel specimens. This sample set comprised two specimens subjected to cadmium treatment, another two exposed to copper, an additional pair treated with nickel, two more subjected to a combined mixture of these metals, and finally, a pair of untreated control specimens labeled as CTRL. We only used two samples for each condition because the number of male specimens we had available was very low, and also because some samples were used to calibrate the analyses before proceeding with this experimentation. To enhance the reliability of our data analysis, the Synthetic Minority Over-sampling Technique (SMOTE) algorithm was employed to generate five synthetic samples, one for each class. Subsequently, a partial least squares discriminant analysis (PLS-DA) algorithm was applied to the gamete dataset, yielding a robust and statistically significant model characterized by a substantial class separation (R2 = 0.999, indicating an excellent fit to the known data, and Q2 = 0.108, suggesting limited predictive capability for unseen data due to likely sample scarcity). This was complemented by cross-validation accuracy = 0.386 and three single latent variables with *p* = 0.581. The visual representation in Figure 1 (Panels A, B, and C) showcases this model’s effectiveness in class discrimination while emphasizing that, since the primary goal is not prediction but rather comprehension and adaptation to existing data, the lower Q2 value could be deemed acceptable. Once again, metabolites possessing a VIP score exceeding 1.5 were identified, as illustrated in Figure 2A, and a heatmap was employed to collectively present the top 25 metabolites derived from the ANOVA analysis (Figure 2B). The red color is employed to signify instances with elevated concentrations, while the blue color is employed to denote those with diminished concentrations, as delineated by the corresponding bar on the VIP score and heatmap graph.

**Figure 3:**
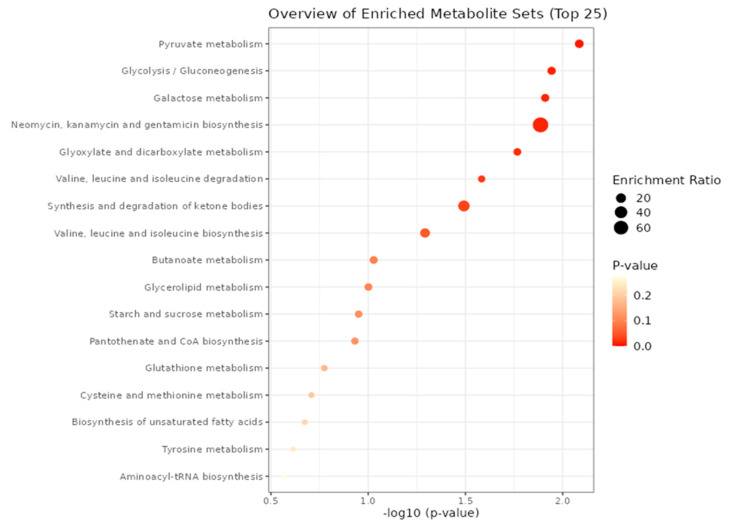
Enrichment analysis using the most relevant metabolites.

Furthermore, we conducted a metabolite-set enrichment analysis, illustrated in Figure 3, using the identified metabolites. This comprehensive analysis unveiled intricate relationships and connections within diverse metabolic pathways and among various metabolites, shedding light on the complex interplay within the metabolic network.

## 4. Discussion

As a result of anthropogenic activities, the levels of pollutants in the marine environment have been increasing over the last few decades, and heavy metals are the major anthropogenic contaminants of coastal waters [33]. Metabolomics is the field of science that characterizes the endogenous and exogenous metabolites within a cell, tissue, or biofluid of an organism in response to external stressors such as disease, exposure to contaminants, or nutritional imbalances [34], and has been applied to the identification of biomarkers for heavy metal exposure. For this reason, it may prove to be an excellent approach to measure the responses of *M. galloprovincialis* to different environmental stresses. In the present study, 248 metabolites were identified in the male gonads of *M. galloprovincialis*, and they were quantified after the exposure of this organism to different metals and to a mixture of these metals. Specifically, the most abundant metabolites found in gonads were eight (lactic acid, N-Methylphenylethanolamine, myo-inositol, acetoacetic acid, glycerol, oxalic acid I, oxalic acid II, and L-valine). After exposure to the different metals tested in this study, the levels of these metabolites varied. The lactic acid amount increased after exposure of mussels to copper and cadmium, compared to the control condition, while it decreased after exposure to nickel and to the mixture of the metals tested. For this metabolite, the literature lacks information regarding effects on the mussel gonadal tissue, but some information is available on the gills tissue of some fishes. For example, an increase of lactate levels has been demonstrated after exposure to copper in silver carp (*Hypophthalmichthys molitrix*), which is considered to result from anaerobic metabolism caused by gill impairment [35]. For similar reasons, the exposure to cadmium also produces an increase of lactate levels in silver carp, for which plasma lactate was significantly elevated after exposure to cadmium [36].

Aspects of the hormonal regulation of gonadal physiology, such as cellular metabolism, are important and have proved interesting to explore. Only information on human and bull gonads is available in more detail. Studies have previously reported the presence of organic acids in human spermatozoa and the seminal plasma of bulls and humans. Organic acids play crucial roles during anabolism by providing C-atom backbones, suggesting that bull spermatozoa have an active energy metabolism [37]. In fact, lactate is a key metabolite that is a fuel for germ cell development and maintains a high rate of protein synthesis in spermatocytes and spermatids [38]. Indeed, lactate infusion into adult cryptorchid testes has been shown to improve spermatogenesis [39]. The dysregulation of male germ cell apoptosis has been associated with the pathogenesis of male infertility [40]. Based on these considerations, the increase in lactate in the gonads of mussels following exposure to copper and cadmium could represent a defense system against germ cell apoptosis, as reported in humans [40]. The decrease in lactate resulting from the mussels’ exposure to nickel and to the metal mixture could presumable cause fertility problems, because nickel inhibits the enzyme lactate dehydrogenase, which catalyzes the reaction that leads from pyruvate to lactate [41], in line with what has been observed in Klinefelter (KS) men, in whom a severe reduction in lactate accumulation within testicular tissue for differentially transcribed lactate dehydrogenase was found [38]. In contrast, myo-inositol (MI) was found to be the most abundant metabolite in the control and cadmium conditions compared to all other exposure conditions, particularly after nickel exposure. MI plays a highly relevant role as a cellular osmolyte, protecting cells from environmental stress, by osmotic compensation and consequent cell fluid and volume balance [42]. In humans, MI is mainly produced by Sertoli cells in response to follicle-stimulating hormone (FSH), and is involved in processes that include the regulation of motility, capacitation, and acrosome reaction of sperm cells [43]. In many mammals, the concentration of myo-inositol in the fluid of the seminiferous tubules is dramatically higher than the levels found in serum. Additionally, in spermatozoa, MI is involved in a variety of transmission mechanisms regulating cytoplasmic calcium levels, capacitation, and mitochondrial function. Most of them have an effect on the reduction of ROS levels and thus on the improvement of sperm motility. In this context, myo-inositol is an important natural compound whose action as an antioxidant molecule has been well documented [44]. One possible explanation for the increase in the level of MI observed after the mussels’ exposure to cadmium could be that this metabolite has protective effects on gonadal damage. In fact, it has been demonstrated in mice that CdCl_2_ induces testicular lesions, increasing iNOS, TNF-α expression, and malondialdehyde (MDA) levels, and lowers glutathione (GSH). MI significantly reduced iNOS, TNF-α expression, and MDA levels, and increased GSH [45]. A similar protective effect could take place in mussels. A similar trend of MI was found for acetoacetic and glycerol; they were found to be present in greater quantities after exposure to cadmium and in the control condition, whereas under all other exposure conditions, there was a decrease in the presence of these metabolites. A metal exposure study using blue mussel *Mytilus edulis* demonstrated that Cd could significantly disturb its lipid metabolism by changing lipid structures and the fatty acid composition [46]. Acetoacetate, a by-product of fatty acid metabolism, is an important source of energy for many organs. The body uses acetoacetate with the help of succinyl-CoA transferase (SCOT). SCOT is ubiquitously expressed in various organs. A novel SCOT-t has now been identified that is specifically expressed in testicular germ cells and spermatozoa. These results indicate that ketone bodies might be involved in sperm movement to allow sperm movement in the female genital tract, but not in the acrosome reaction, and that the enzyme SCOT-t identified in sperm mitochondria might play an important role in sperm activity, resulting in male infertility when its function is disabled [47]. The exposure to cadmium produces an increase of acetoacetic acid. It is reported in the literature that acetoacetic acid produces a decrease of reduced glutathione (GSH) [48]. GSH deficiency causes an increased susceptibility to oxidative stress. As a matter of fact, cadmium is one of the heavy metals which produce oxidative damage [49]. Glycerol is potentially a substrate for gluconeogenesis, glycolysis, lipogenesis, and glyceroneogenesis [50]. A possible defensive mechanism which explains the increase of glycerol in the gonads of mussels following cadmium exposure could be that the high osmolarity glycerol pathway and the cell wall integrity pathway are all essential for the defense against the cadmium-induced toxicity, including the elevated ROS and cell death levels induced by cadmium [51]. Oxalic acid was more present after exposure to cadmium and copper. The most striking chemical impact of oxalic acid is its strong chelating ability with multivalent cations, and it is available as a natural antioxidant, playing an important role in the natural and artificial preservation of oxidized materials [52]. The alterations observed in the level of oxalic acid could be indicative of the unbalance of the redox system. As reported in the literature, oxalic acid correlated significantly with the number of sperm head anomalies, the pH value, and the age of the donors, and correlated negatively with the volume of ejaculate [53]. In particular, some researchers cited oxalic acid as a regulator of intracellular pH; therefore, an alteration of this parameter could play an important role in fertility capability [54]. Finally, L-valine (Val) was found to be more present after exposure to cadmium and copper, compared to the control condition. High levels of Val cause histological damage on testes, suggesting a reduction in the percentage of spermatozoa that may interfere with the process of cell division, which may have an effect on the process of spermatogenesis [55,56]. Valine biosynthesis was found to be altered, in line with the results of the study conducted to evaluate the toxicological effects of cadmium on deep-sea mussel *Gigantidas platifrons* by a combined proteomic and metabolomic approach [57]. In another study, performed with an exposure of mussels to BPA, female mussel gonads had lower levels of branched chain amino acids, including valine, than those in male mussel samples. These metabolic differences indicated that there exist intrinsic (gender-specific) biological differences between male and female mussel gonads. These gender differences at the metabolite level could result in gender-specific responses to toxicant exposures [58]. A large number of heavy metals have been shown to be toxic to the reproductive system. However, infertility in invertebrates is poorly understood and, in particular, molecular, cellular, and toxicological studies are limited. In the present work, we show preliminary data on the effect of certain metals and their mixture on metabolites in gonads. Nevertheless, we have already demonstrated the negative effects on the properties of protamine-like proteins (PLs), which represent the major basic protein component of sperm chromatin, after the exposure of M. galloprovincialis to three individual metal chlorides and their mixture tested in the present work and under the same exposure conditions [49]. Under all exposure conditions, relevant changes were observed in the electrophoretic pattern, by AU-PAGE and SDS-PAGE, and in the fluorescence spectroscopic measurements of the PLs. In addition, changes in the DNA binding of these proteins were observed by means of the electrophoretic mobility shift assay (EMSA) and by means of their release from sperm nuclei [49]. There was also evidence of increased accessibility of micrococcal nuclease to sperm chromatin. This was confirmed after staining the spermatozoa with toluidine blue. Moreover, morphology analyses indicated severe gonadal damage, also confirmed by increased PARP expression using Western blotting and sperm DNA fragmentation using the comet assay. The expression of the stress genes GST, HSP70, and MT10 in the gonads was also found to be altered [49]. These results suggest that these metals may have a negative impact on the reproductive fitness of *M. galloprovincialis* [49]. Moreover, we also demonstrated that the gonadal tissue has a metal accumulation ability comparable to the gills, despite it not being a tissue used for filtration [59]. For the eight most abundant metabolites found in the gonads in the present work, an opposite trend was observed after exposure to copper and cadmium compared to nickel. This could depend on the dose of nickel chloride used in this experiment. In fact, in our work, after exposing the mussels to three doses of nickel chloride, 5, 15, and 35 µM, we obtained the lowest level of π-gst expression precisely at 15 µM, denoting a possible hormesis effect [60]. Hormesis is a dose/response effect which is characterized by a biphasic effect. A number of organisms/biological systems, when exposed to a variety of stimuli, exhibit opposite responses dependent on the dose. Several examples of hormesis effects are provided in the literature, such as the cadmium-induced effect on growth and photosynthetic performance in a hyperaccumulator, Lonicera japonica Thunb. Hormesis is seen as an adaptive function. It has been argued by several authors that hormesis, which can be observed in all living organisms, plants included, may be due to physiological state changes or the modification of regulatory mechanisms caused by external agents. Presumably the course of this stress gene could mirror the course of metabolites found in the gonads. In conclusion, the results obtained in the present work do not currently allow us to precisely determine the specific role of each metal in the metabolite pathway and its function. This represents only preliminary work, and further investigations will therefore be extremely necessary to link the metabolomic data to the alterations found at the molecular level. Certainly, other metabolomic or proteomic pathways might be influenced. In order to understand the biological mechanisms triggered by specific chemical compounds, the combination of metabolomics with information on the presence of xenobiotics in biological samples (xenometabolome) and other molecular information obtained from omics technologies (e.g., transcriptomics, proteomics) is crucial, both in exposure studies and in the development of adverse outcome pathways.

## 5. Conclusions

This ground-breaking research is one of the first investigations into the metabolic changes in the gonadal tissue of *M. galloprovincialis* upon exposure to heavy metals, also considering that the gonad is not a filter tissue. All metabolites were more abundant in the gonads after exposure to copper and cadmium compared to non-exposure conditions, nickel, and a mixture of the three metals tested. We may think that exposure to copper and especially cadmium may pose problems for the reproductive health of *M. galloprovincialis*, since some of the metabolites have antioxidant activity and others, when present in high concentrations, inhibit spermatogenesis, sperm motility, and induce tissue damage. The increased metabolites observed in this study, particularly with cadmium, demonstrate how the presence of these metals can underlie physiological and metabolic problems. While the increase in these metabolites after exposure to cadmium may represent reproductive problems, in some cases, their increase is correlated with a protective effect. Therefore, the balance between the positive and negative effects is very important in determining the actual effects on this organism. Given the complexity of the metabolic pathways even in a simple organism, this work has the obvious limitation that this complexity poses to the proper understanding of these pollutant–organism interactions, also considering that this is an organism that has been little-studied in this regard and with little scientific literature. Certainly, this study is only a first step to begin to understand what direction can be pursued to arrive at a better understanding. Therefore, further studies with greater molecular resolution are needed to fully understand the implications of these initial changes.

## Data Availability

The data presented in this study are available on request from the corresponding author. The data are not publicly available due to better management of data sharing and give support for better understanding of raw data.
